# In Silico Insights into the SARS CoV-2 Main Protease Suggest NADH Endogenous Defences in the Control of the Pandemic Coronavirus Infection

**DOI:** 10.3390/v12080805

**Published:** 2020-07-26

**Authors:** Annamaria Martorana, Carla Gentile, Antonino Lauria

**Affiliations:** Dipartimento di Scienze e Tecnologie Biologiche Chimiche e Farmaceutiche, University of Palermo, Viale delle Scienze–Ed. 17, I-90128 Palermo, Italy; annamaria.martorana@unipa.it (A.M.); carla.gentile@unipa.it (C.G.)

**Keywords:** coronavirus, COVID-19, SARS-CoV-2 main protease, DRUDIT web service, molecular docking, HIV-protease, NADH

## Abstract

COVID-19 is a pandemic health emergency faced by the entire world. The clinical treatment of the severe acute respiratory syndrome (SARS) CoV-2 is currently based on the experimental administration of HIV antiviral drugs, such as lopinavir, ritonavir, and remdesivir (a nucleotide analogue used for Ebola infection). This work proposes a repurposing process using a database containing approximately 8000 known drugs in synergy structure- and ligand-based studies by means of the molecular docking and descriptor-based protocol. The proposed in silico findings identified new potential SARS CoV-2 main protease (M^PRO^) inhibitors that fit in the catalytic binding site of SARS CoV-2 M^PRO^. Several selected structures are NAD-like derivatives, suggesting a relevant role of these molecules in the modulation of SARS CoV-2 infection in conditions of cell chronic oxidative stress. Increased catabolism of NAD(H) during protein ribosylation in the DNA damage repair process may explain the greater susceptibility of the elderly population to the acute respiratory symptoms of COVID-19. The molecular modelling studies proposed herein agree with this hypothesis.

## 1. Introduction

The novel coronavirus (CoV) SARS-CoV-2, also known as 2019-nCoV, is the pathogen that has caused the present pandemic (known as 2019-nCoV disease or COVID-19). In late December 2019, the disease was declared for the first time in China when a conspicuous number of patients presenting viral pneumonia with severe acute respiratory syndrome (SARS) were observed in the city of Wuhan [1]. According to the situation report n.67 of the World Health Organization (WHO, http://www.who.int), the number of worldwide SARS-CoV-2 infected patients on 14 May 2020 was 4,258,666 with 294,190 deaths, fixing the risk assessment as “very high” at the global level. The CODIV-19 infection causes typical para-flu symptoms, such as dry cough, fever, headache, dyspnoea, and pneumonia, which may degenerate into progressive severe respiratory failure due to alveolar damage, leading to death in some cases [1].

According to the WHO, the SARS-CoV-2 virus infects people of all ages. However, in elderly people (over 60 years old), especially those with previous pathologies (such as chronic respiratory diseases, diabetes, cardiovascular diseases, and cancer), SARS-CoV-2 infection leads to more serious clinical symptoms that almost always involve intensive care. In Italy, the percentage of COVID-19 deaths in 60-year-old people is greater than 95% of the total COVID-19 deaths.

Currently, WHO is focusing attention on the following COVID-19 experimental therapies: antiviral drugs, including lopinavir/ritonavir, used for HIV infection; remdesivir, belonging to the class of nucleotide analogues, used for Ebola virus disease; anti-malaria molecules, including chloroquine and hydroxychloroquine; and a monoclonal antibody against IL-6 approved for chronic inflammatory diseases [2].

To contain the infection, the scientific community suggests strong social containment measures and active development of a vaccine, which may be available within the next 18 months.

For the development of new pharmacological therapies, the drug repurposing approach [3], which assigns new therapeutic uses to known drugs, represents a promising method to bypass the long-term process of pharmacokinetics and toxicological clinical studies. Therefore, this approach has great potential in an emergency situation similar to the present situation.

SARS-CoV-2 is a human β coronavirus originating from bats, crossing snake to human [4]. β coronaviruses have an enveloped coating and present an ssRNA positive-strand. The SARS-CoV-2 genome has approximately 80% sequence identity to SARS-CoV and 50% sequence identity to MERS-CoV (Middle East respiratory syndrome coronavirus) [5]. In addition, homology modelling shows a deep similarity of the receptor–binding domain of SARS-CoV-2 with SARS-CoV, which recognizes the ACE2 receptor in human cells for infection [6]. In February 2020, the crystallized image of the main protease (M^PRO^), chymotrypsin-like protease (3CL^PRO^), of bat SARS-CoV-2 (PDB Code 6LU7) in complex with a peptidomimetic inhibitor (N3) was communicated to the scientific community [7]. In coronaviruses, 3CLpro is a cysteine catalytic enzyme, which cleaves the C-terminus of the polyprotein of the SARS coronavirus replicase at 11 sites. The selective inhibition of the virus main protease may interfere with the construction of the RNA replicase, blocking the replication of the RNA genome from the virus RNA template, ultimately halting the infection of human cells [8].

The present study aimed to contribute information to combat the COVID-19 pandemic. In this work, a large database containing approximately 8000 structures of well-known drugs (approved, experimental, and investigational) [9] was analysed with a virtual screening protocol to repurpose [3] their therapeutic use as selective inhibitors of the SARS CoV-2 main protease (COVID-19 M^PRO^). Given the urgent need to find efficient strategies for mitigating the effects of the pandemic, computational studies may rationalize the experimental clinical strategies currently administered in COVID-19 patients and may suggest different drugs to cure infected patients.

## 2. Materials and Methods 

### 2.1. Structure-Based Studies

The ligands and protein–ligand complex used for the in silico studies were prepared as detailed below. 

#### 2.1.1. Ligand Preparation

The default setting of the LigPrep tool implemented in Schrödinger’s software (Version 2017-1) was used to prepare the ligands for docking [10]. All possible tautomers and the combination of stereoisomers were generated at pH 7.0 ± 0.4 using the Epik ionization method [11]. Energy minimization was subsequently performed using the integrated OPLS 2005 force field [12].

#### 2.1.2. Protein Preparation

The crystal structure of COVID-19 M^PRO^ in complex with ligand 13b (PDB id 6Y2F) [13] was downloaded from the Protein Databank [14]. The cocrystal ligand, covalently bonded to C145, was treated by breaking the covalent bond and filling in open valence. The Protein Preparation Wizard of Schrödinger software was subsequently employed for further preparations of the protein structure using the default settings [15]. Bond orders were assigned, and hydrogen atoms, as well as protonation of the heteroatom states were added using the Epik-tool (with the pH set at biologically relevant values, i.e., at 7.0 ± 0.4). The H-bond network was then optimized. The structure was subjected to a restrained energy minimization step (the RMSD of the atom displacement for terminating the minimization was 0.3 Å), using the Optimized Potentials for Liquid Simulations (OPLS) 2005 force field [12].

#### 2.1.3. Docking Validation

Molecular docking was performed by the Glide program [16,17,18]. The receptor grid preparation was performed by assigning the original ligand (**13b**) as the centroid of the grid box. The generated 3D conformers were docked into the receptor model using the Extra Precision (XP) mode as the scoring function. A total of 5 poses per ligand conformer were included in the post-docking minimization step, and a maximum of 2 docking poses were generated for each ligand conformer. The proposed docking procedure was validated by the re-dock of the crystallized 13b within the receptor-binding pockets of 6Y2F by Glide covalent docking. The results obtained were in good agreement with the experimental poses, showing an RMSD of 0.75.

#### 2.1.4. Induced Fit Docking

Induced fit docking simulation was performed using the IFD application as available [19,20] in the Schrödinger software suite [21], which has been demonstrated to be an accurate and robust method to account for both ligand and receptor flexibility [22].

The IFD protocol was performed as follows [23,24]: The ligands were docked into the rigid receptor models with scaled down van der Waals (vdW) radii. The Glide Extra Precision (XP) mode was used for the docking, and twenty ligand poses were retained for protein structural refinements. The docking boxes were defined to include all amino acid residues within the dimensions of 25 Å × 25 Å × 25 Å from the centre of the original ligands. The induced-fit protein–ligand complexes were generated using Prime software [25,26]. The 20 structures from the previous step were submitted to side chain and backbone refinements. All residues with at least one atom located within 5.0 Å of each corresponding ligand pose were included in the refinement by Prime. All the poses generated were then hierarchically classified, refined, and further minimized into the active site grid before being finally scored using the proprietary GlideScore function defined as follows in Equation (1):

GScore = 0.065 × vdW + 030 × Coul + Lipo + Hbond + Metal + BuryP + RotB + Site(1)

where vdW is the van der Waals energy term, Coul is the Coulomb energy, Lipo is a lipophilic contact term that rewards favourable hydrophobic interactions, Hbond is an H-bonding term, Metal is a metal-binding term (where applicable), BuryP is a penalty term applied to buried polar groups, RotB is a penalty for freezing rotatable bonds, and Site is a term used to describe favourable polar interactions in the active site.

Finally, the IFD score (IFD score = 1.0 Glide_Gscore + 0.05 Prime_Energy), which accounts for both the protein–ligand interaction energy and total energy of the system, was calculated and used to rank the IFD poses. More negative IFDscore values indicated more favourable binding.

### 2.2. Biotarget Finder Module (DRUDIT)

The refined selection of suitable COVID-19 M^PRO^ inhibitors was performed through the module Biotarget Finder as available on the https://www.drudit.com webserver [27]. The tool allows predicting the binding affinity of candidate molecules versus the selected biological target. The template of the biological target was built by using the top scored structures obtained by molecular docking (see above). The resulting structures were uploaded to the webserver and elaborated to build the COVID-19 M^PRO^ template.

The drug database was submitted to the Biological Predictor module by setting the DRUDIT parameters, N, Z, and G, using the crystallized structure of **13b**. In detail, the tuning was executed by changing the DRUDIT parameters (N, 200–500–1000; Z, 50–100; G, a–b–c). Table 1 shows the results obtained by submitting the reference Compound **13b** to the Biotarget Affinity Module focused on the COVID-19 M^PRO^ template.

## 3. Results and Discussion

**Figure 1:**
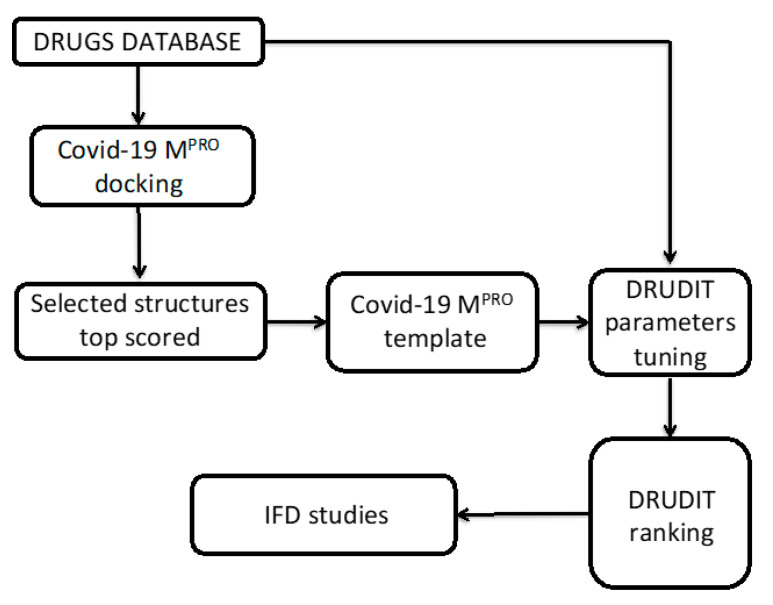
Flowchart of the proposed protocol. IFD, induced fit docking.

In this study, we proposed an in silico protocol to provide insights into the mechanism of action that governs the activity of the SARS-CoV-2 main protease and to propose new potential inhibitors. Given the emergency, we neglected the routine and time-intensive preclinical investigations to directly explore a database of known drugs [9]. A schematic flowchart of the adopted protocol is depicted in Figure 1.

**Figure 2:**
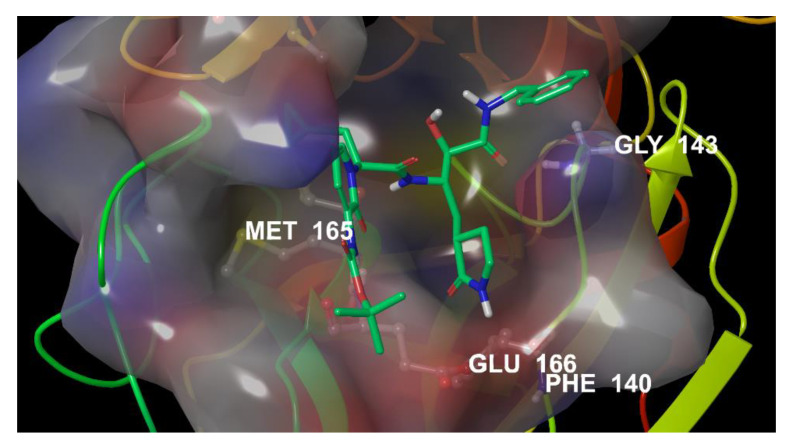
3D binding site of SARS-CoV-2 M^PRO^ in complex with inhibitor **13b** (PDB id 6Y2F).

As a first step, we performed molecular docking studies on the entire set of known drugs to analyse their fit in the catalytic active site of the recently identified SARS-CoV-2 M^PRO^ (PDB id: 6Y2F) [13] as reported in the Materials and Methods Section. Figure 2 shows the 3D binding active site of SARS-CoV-2 M^PRO^ co-crystallized with the native inhibitor **13b** covalently bonded to C145. 

The ligand binds to the enzymatic catalytic cleft of the protease located between domains I and II. The 3D binding site representation (Figure 2) highlights interactions with the amino acid residues involved in the inhibition mechanism, such as M49, M165, E166, H164, F140, G143, and the catalytic C145. It is noteworthy that the hydrogen bonds between the pyridone moiety of the ligand and E166 of chain A, which rules the catalytic activity, drive the SARs-CoV-2 main protease to adopt an inactive conformation [13]. 

The resulting best docked molecules were selected based on a docking score cut-off of -7.0 Kcal/mol (Supplementary Material, S1) and used to build the template of SARS-CoV-2 M^PRO^. The model of SARS-CoV-2 M^PRO^ was integrated in the DRUDIT (DRUgs Discovery Tools) webservice, an open access virtual screening platform recently developed by our group (https://www.drudit.com) [27], based on our experience with molecular descriptors [28,29]. The DRUDIT protocol optimizes/tunes the model for input biological targets, starting from known modulators to setting up the DRUDIT N, Z, and G parameters. In detail, the N parameter defines the number of dynamically selected molecular descriptors, capturing the relevance of each molecular descriptor Di to the biological target set. Z is the maximum percentage of unavailable values (zeros) for each molecular descriptor, and G denotes the Gaussian smoothing function. In the DRUDIT Biotarget Predictor tool, the DRUDIT affinity score (DAS) is assigned by weighting the scores (the sum of all values divided by the number of molecular descriptors), which are correlated to the chosen G function only for the molecular descriptor selected according to the Z and N parameters.

Therefore, to set DRUDIT Biotarget Predictor tool parameters, the co-crystallized native ligand of SARS-CoV-2 M^PRO^ (Figure 2) was used (PDB id 6Y2F) [13]. The tuning was executed with the parameters spanning within the ranges 200 < N < 800 and 50 < Z < 100, as well as considering a, b, or c for the G function. The N, Z, and G parameters, corresponding to the highest values of DAS for Compound **13b**, were selected (Materials and Methods). 

Generating the SARS-CoV-2 M^PRO^ template and setting the N, Z, and G parameters, as above reported, allowed evaluation of the in silico affinity of the selected drugs to SARS-CoV-2 M^PRO^ through the DRUDIT Biological Affinity tool. The features of the ligand-based approaches based on molecular descriptors allowed evaluation of the topological, thermodynamic, and charge-related characteristics of the ligands. Thus, two complementary standpoints in the evaluation of the binding capability (ligand- and structure-based) covered all the interaction aspects in the ligand-target complex.

The DAS values of the selected molecules (Supplementary Material, S2) with the highest predicted biological affinity for SARS-CoV-2 M^PRO^ are shown in Table 2.

All selected molecules were processed by induced fit docking (IFD) calculations to better investigate the structural interactions and the binding poses with the cysteine catalytic active site of SARS-CoV-2 M^PRO^ (Table 2). Most ligands interact with the pivotal amino acid residues, which have a central role in the catalytic enzyme process of the protease. The non-covalent interactions with F140 and G143, as well as C145 and H164 (to a greater extent) are frequent. 

**Figure 3:**
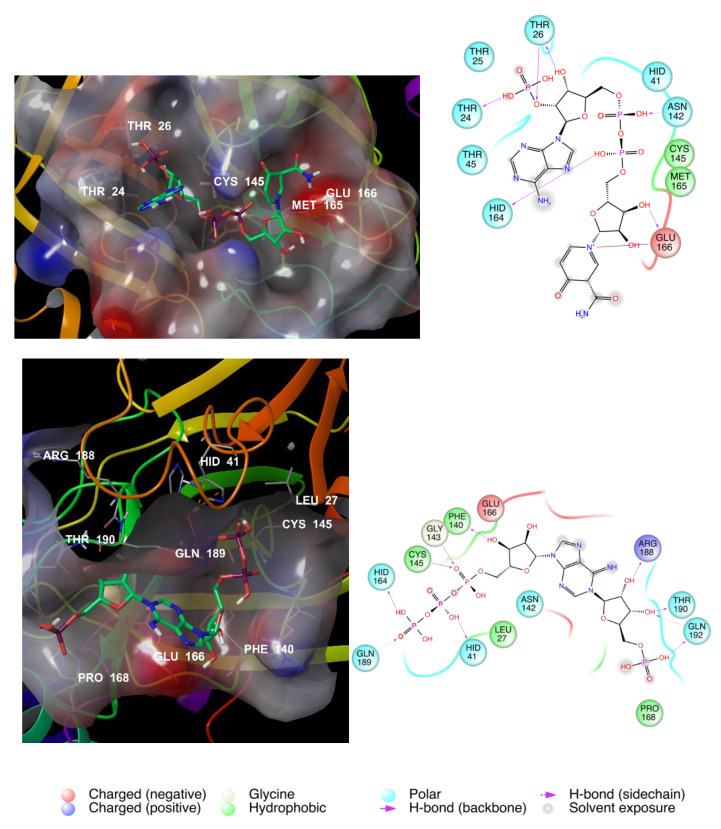
Binding of the best scored compounds (ID 49867432, top image; and ID 16019963, bottom image) into the COVID-19 M^PRO^ active site (left) and amino acid maps (right).

Figure 3 shows the best scored molecules, namely ID 49867432 and ID 16019963. The two NADH-like molecules are deeply buried in the cleft of the substrate-binding pocket, and in line with the observation for the co-crystallized ligand **13b**, the two selected NADH-like ligands interact with the recurrent amino acids H164, F140, G143, and C145. In particular, hydrogen bonds with the ribose moiety and E166 avoid the access of protein promoter B and induce the enzyme to adopt an inactive conformation. 

### 3.1. Repurposing of Known HIV Protease Inhibitors

**Figure 4:**
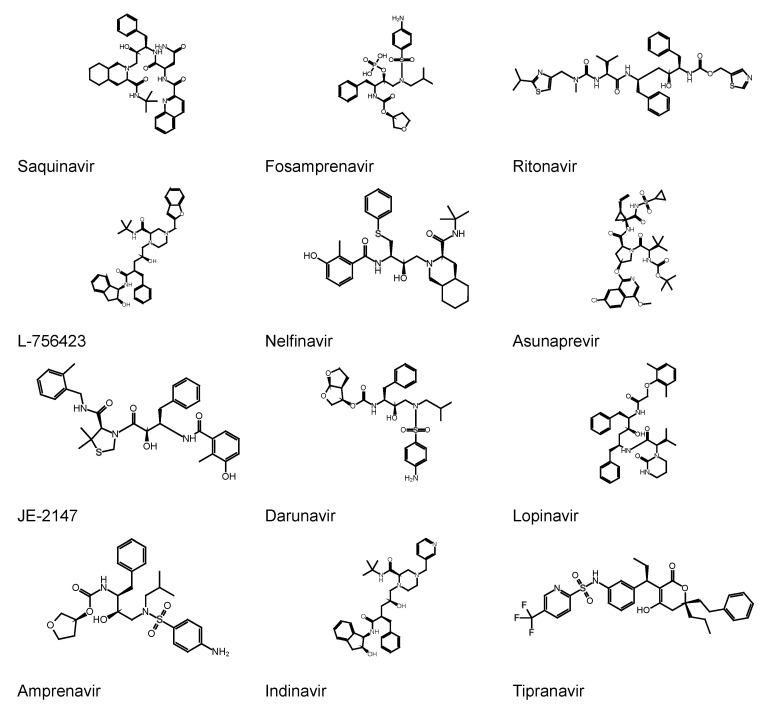
HIV protease inhibitors.

Given the emergency and the pandemic expansion of the virus, clinicians have experimentally used known HIV protease inhibitors for the therapeutic treatment of SARS-CoV-2 patients. Thus, to test the DRUDIT SARS-CoV-2 M^PRO^ model and rationalize the repurposing of HIV antiviral drugs, a set of known HIV-1 protease inhibitors (Figure 4) was studied against SARS-CoV-2 M^PRO^ and HIV-1 protease models, submitting them to the DRUDIT Biotarget Finder Module (Table 3).

The results indicated that only a few known HIV protease inhibitors have a good affinity to SARS-CoV-2 M^PRO^. In particular, the best scored compound is fosamprenavir followed by darunavir, amprenavir, and tipranavir. 

**Figure 5:**
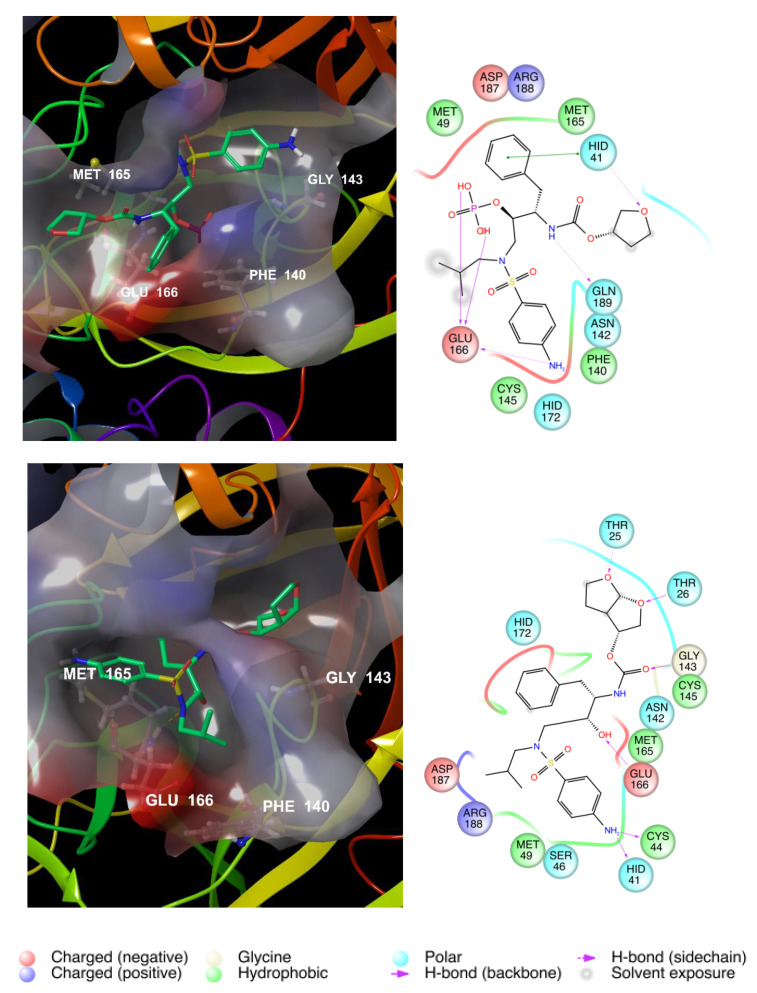
Fosamprenavir (top) and darunavir (bottom) binding to the COVID-19 M^pro^ active site.

To analyse the structural features that enhance the binding capability of the best scored compounds, IFD analysis was performed (Table 4). The binding poses of the top-scored drugs in the catalytic active site of SARS-CoV-2 M^PRO^ are illustrated in Figure 5. 

G143, F140, M165, and C145 are the recurring amino acids that actively interact in the ligand-protein binding, highlighting a good affinity for the protease. The interactions with the key amino acid, E166, are pivotal.

### 3.2. NAD as a Potential Modulator of COVID-19 M^PRO^

Further analysis of the obtained results (Supplementary Material, S2) by considering recurrent scaffolds showed that ten of the selected structures are NAD derivatives.

**Figure 6:**
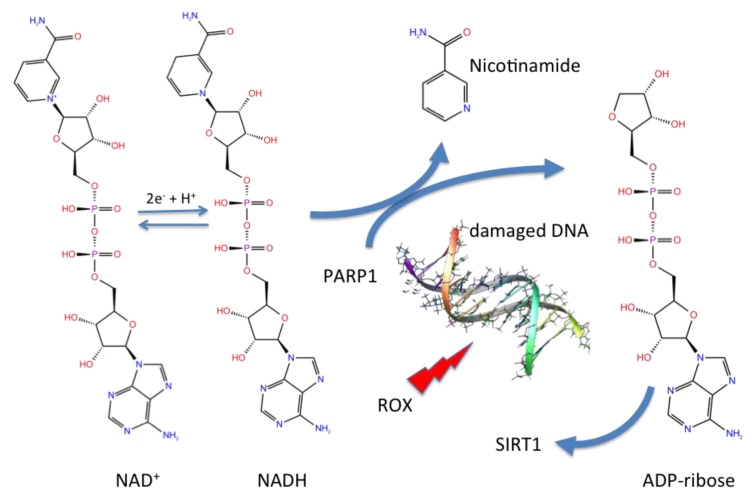
Nicotinamide adenine dinucleotide (NAD) redox equilibrium and its role in DNA repair. DNA damage activates the PARP1 enzyme, resulting in consumption of nicotinamide adenine dinucleotide (NAD^+^) as a substrate to produce ADP-ribose units.

The nicotinamide adenine dinucleotide (NAD) redox co-factor is involved in several cellular redox reactions, in which it acts as an electron source or acceptor, in a continuous turnover between the oxidized form (NAD^+^) and the reduced form (NADH), as depicted in Figure 6. 

Moreover, this ubiquitous co-factor is also involved in defence reactions to oxidative damage, in particular in DNA repair involving the poly(ADP-ribose) polymerase-1 (PARP) DNA nick sensor. In the PARP-catalysed reaction, NAD supplies ADP-ribose units and is then catabolized (Figure 6).

Under conditions of moderate DNA damage, activation of PARP-1 involves an NAD loss easily recovered from the cellular biosynthetic activity. However, excessive DNA oxidative damage and overactivation of PARP1 increases NAD catabolism, resulting in adverse effects in the energy production process (Figure 6). 

A similar situation can arise in conditions of increasing oxidative stress, such as during ageing. Redox unbalance is indeed one of the main drivers of the ageing process cause and/or consequence of mitochondrial dysfunction [30]. Therefore, it is likely that the associated oxidative stress during aging not only chronically consumes the endogenous antioxidant defences, but also activates repair processes that cannot be supported by cells in the long run. 

For the first time, Massudi and colleagues associated increased NAD catabolism in human tissue to hyperactivation of PARP due to oxidative damage during ageing [31]. Several subsequent studies have demonstrated that NAD levels decrease with age, promoting several ageing-associated metabolic and neurodegenerative diseases. Conversely, dietary supplementation with NAD exhibits beneficial effects against ageing and ageing-associated diseases (Yaku K, 2018) [32].

Analysis of the statistical report of the mortality due to COVID-19 showed that age has a crucial role in the mortality of the virus infection (Table 5).

Based on these observations and in view of the studied high affinity of several NAD derivatives towards the COVID-19 M^pro^ active site, we hypothesized a potential role of higher NAD levels in the young population in protecting against SARS-CoV-2 infection.

**Figure 7:**
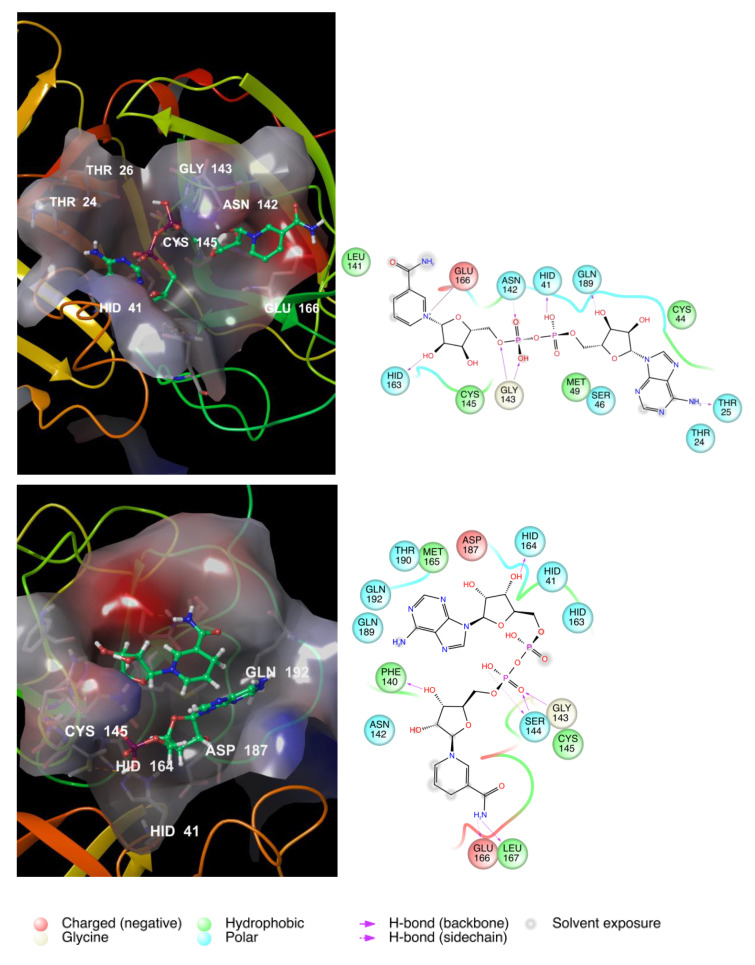
3D binding modes of NAD^+^ and NADH (left) and amino acid maps (right).

Thus, to test this hypothesis, we submitted NAD+/NADH to the IFD and DRUDIT protocols. The results showed high biological affinity of NADs, both reduced and oxidized forms, for the SARS CoV-2 main protease (Table 6 and Figure 7). The amino acid maps showed several non-covalent interactions with the above highlighted crucial AAs (M165, F140, E166, and G143, as well as C145 and H164 to a greater extent). The binding site of COVID-19 M^PRO^ adapts the cavity to both NAD species (charged and uncharged) by changing its shape. The charged NAD form is buried in the cavity, which keeps and stretches the molecule as in the palm of a hand. The NADH uncharged form fits the cavity as a claw kept by a baseball glove, also interacting with all crucial AA residues. 

Associated with the oxidative stress of cells and the changing levels of NAD during human life, NAD(H) molecules may modulate Sars-CoV-2 M^PRO^ activity. In this light, our hypothesis may explain the greater frequency of the most severe respiratory symptoms in elderly COVID-19 patients.

Although the tissue distribution of host receptors is generally consistent with the tropisms of viruses [33] and the lung receptiveness to SARS-CoV-2 can be justified by the high expression of ACE2 receptors in human airway epithelia and lung parenchyma [34], the presence of ACE2 is not sufficient to make host cells susceptible to infection. Indeed, some ACE2-expressing cells fail to be infected by SARS-CoV [35]. Therefore, other parameters must also be considered to justify the specific virus tropism.

Airway cells are the most exposed cells to oxidative damage. The alveolar compartment is permanently subjected to potential pro-oxidative agents derived from inhaled air pollutants [36]. Aged airway cells are subjected to physiological oxidative insults with consequent increases in chronic conditions of oxidative stress in the elderly population. Therefore, lower NAD levels in elderly subjects may result in more hazardous consequences for the respiratory tract compared to other body systems, which may be of crucial importance in the fight against COVID-19 infection. If this hypothesis is confirmed, the scenario for the future control of pandemic spread should be focused on the use of foods and/or food supplements rich in NAD(H) and its derivatives.

## 4. Conclusions and Perspectives

The proposed in silico findings, as performed by a mixed ligand- and structure-based protocol, identified new potential SARS CoV-2 M^PRO^ inhibitors that fit into the catalytic binding site of the protease. Several selected structures are NAD or NAD-like derivatives, suggesting a relevant role of these molecules in the modulation of SARS CoV-2 infection in conditions of cell chronic oxidative stress. The comparison of the IFD results obtained for the repurposed drugs and NADH-NAD^+^ demonstrated that NADs are the molecules with the best fit in the SARS CoV-2 M^PRO^ cavity. 

Increased catabolism of NAD(H) during protein ribosylation in the DNA damage repair process may explain the greater susceptibility of the elderly population to acute respiratory symptoms of COVID-19.

Our results suggested that damaged redox homeostasis of cells, especially in tissues for which SARS-CoV-2 shows greater tropism, may contribute to a particularly serious form of COVID-19. In addition, the inflammation process due to virus infection contributes to exacerbating the oxidative cell damage in a self-amplified manner [37].

Despite the increased incidence of COVID-19 infections in the elderly population and the chronic state of redox imbalance in age-related decline, the use of antioxidant molecules to control SARS symptoms in COVID-19 patients has not been explored. Despite the increased popularity of vitamin C-based supplements coinciding with the explosion of the COVID-19 pandemic, no data have demonstrated that this antioxidant vitamin, also known for its immune-modulatory effects, is useful in COVID-19 patients or to prevent infection. Therefore, considering the important role of NAD metabolism in oxidative stress related to ageing, our findings may corroborate the hypothesis that vitamin C, or in general antioxidant agents, can be useful in the prevention of COVID-19.

## Figures and Tables

**Table 1:** DRUDIT parameter tuning. The selected values are indicated in bold.

Z	D	G		
		a	b	c
**50**	200	0.79	0.66	0.56
	500	0.72	0.54	0.47
	1000	0.47	0.37	0.47
**100**	**200**	**0.85**	0.77	0.69
	500	0.75	0.59	0.52
	1000	0.59	0.45	0.39

**Table 2:** IFD and biotarget DRUDIT affinity scores (DAS) for the selected molecules.

ID	Prime Score	XP Docking Score	IFD Score	SARS CoV−2 M^pro^ DAS
3730	−10,682	−15.09	−549.2	0.583
5884	−11,035	−13.05	−564.8	0.91
5885	−10,967	−13.15	−561.5	0.917
16500	−10,813	−13.66	−554.3	0.89
23700	−10,937	−15.89	−562.7	0.863
123926	−10,797	−12.10	−551.9	0.973
163884	−10,976	−11.52	−560.3	0.957
165491	−10,851	−13.16	−555.7	0.983
170119	−10,720	−12.63	−548.6	0.917
183797	−10,712	−10.74	−546.3	0.843
445888	−10,770	−14.55	−553.0	0.867
446724	−10,803	−14.81	−554.9	0.897
447657	−10,740	−11.19	−548.2	0.863
448108	−11,130	−13.91	−570.4	0.817
448209	−10,825	−14.53	−555.8	0.9
449129	−10,746	−13.41	−550.7	0.807
449366	−10,687	−13.08	−547.4	0.877
4369128	−10,898	−11.97	−556.9	0.9
5281793	−10,709	−15.15	−550.6	0.893
5288989	−10,974	−12.10	−560.8	0.857
5289104	−10,805	−12.48	−552.8	0.99
5289382	−11,615	−12.13	−592.9	0.967
5289437	−116,960	−11.65	−596.4	0.897
6323200	−11,664	−12.84	−596.0	0.723
9875516	−11,712	−12.69	−598.3	0.733
16019963	−11,660	−16.08	−599.1	0.767
17754101	−11,663	−13.05	−596.2	0.94
49867432	−11,777	−13.52	−602.4	0.893

**Table 3:** Biotarget affinity scores (DAS) of known HIV inhibitors against COVID-19 M^PRO^ and HIV-1 protease DRUDIT models.

Drug	SARS CoV-2 M^pro^ (DAS)	HIV-1 Protease (DAS)
Amprenavir	0.836	0.538
Asunaprevir	0.446	0.696
Darunavir	0.841	0.844
Fosamprenavir	0.868	0.763
Indinavir	0.463	0.901
JE-2147	0.784	0.88
L-756423	0.444	0.89
Lopinavir	0.457	0.910
Nelfinavir	0.506	0.907
Ritonavir	0.463	0.881
Saquinavir	0.475	0.898
Tipranavir	0.818	0.756

**Table 4:** IFD results of the best-scored HIV antiviral molecules.

Drug	XP Docking Score	Prime Score	IFD Score
Fosamprenavir	−10.80	−117,070	−596.2
Darunavir	−9.45	−11,631	−591.1
Tipranavir	−8.32	−11,615	−589.1
Amprenavir	−10.48	−11,601	−590.5

**Table 5:** Age dependence of NAD and deaths due to COVID-19.

Age	0–1	30–50	51–70	>71
NAD (ng NAD/mg protein) mean ± SEM	8.54 ± 1.55	2.74 ± 0.41	1.06 ± 0.15	1.08 ± 0.19
# of COVID-19 deaths (in %)	0	3	11	86

**Table 6:** DRUDIT and IFD output results for NAD^+^ and NADH.

cpd	XP Docking Score	Prime Score	IFD Score	SARS CoV−2 M^pro^ (DAS)
NAD^+^	−13.15	−11,628	−594.6	0.98
NADH	−12.40	−11,682	−596.5	0.96

## Supplementary Materials

The following are available online at https://www.mdpi.com/1999-4915/12/8/805/s1: S1, selected structures by molecular docking; S2, selected structures by the Biotarget Finder Module (http://www.drudit.com).
