# Supplementary material for: In Silico Insights into the SARS CoV-2 Main Protease Suggest NADH Endogenous Defences in the Control of the Pandemic Coronavirus Infection
Source: Viruses. 2020 Jul 26;12(8):805. doi: 10.3390/v12080805 (PMC7472248; doi:10.3390/v12080805)
Supplement: Supplementary file 1 [file viruses-12-00805-s001.zip › Supplementary_Material-S1.pdf]

Research Article

In silico insights on SARS Cov-2 Main Protease suggest NADH endogenous defences in the control of the pandemic coronavirus infection

Annamaria Martorana, Carla Gentile, Antonino Lauria*
Dipartimento di Scienze e Tecnologie Biologiche Chimiche e Farmaceutiche - University of Palermo, Viale delle Scienze – Ed. 17 - I-90128 Palermo, Italy.
*Correspondence: antonino.lauria@unipa.it; Phone +39 091238-96818

Supplementary material S1

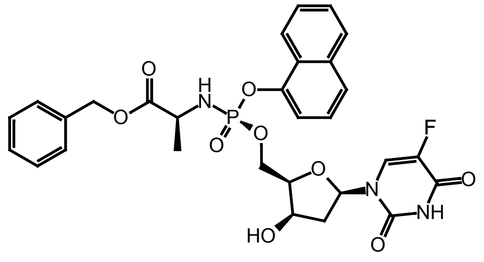

53373585
-9.547

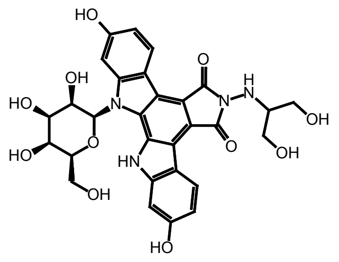

9808998
-9.457

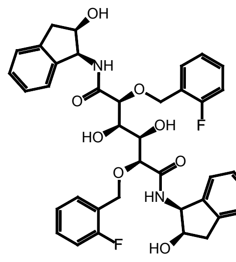

445306
-9.41

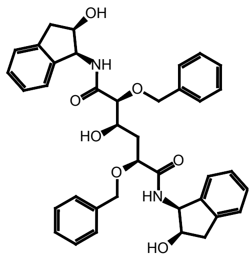

444974
-9.392

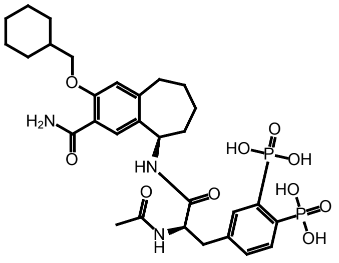

445561
-9.34

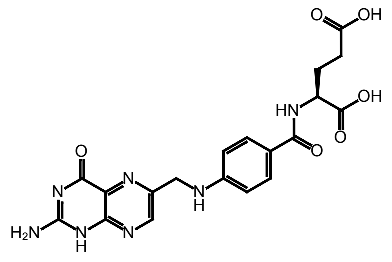

6037
-9.288

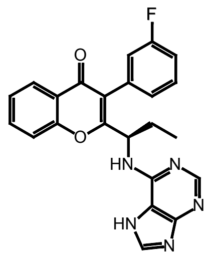

86291103
-9.269

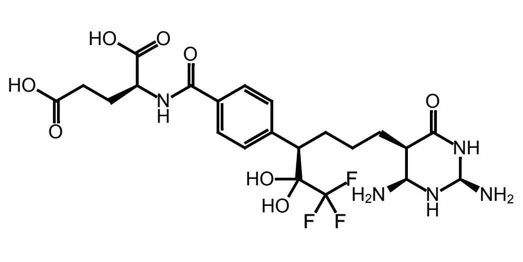

131704252
-9.254

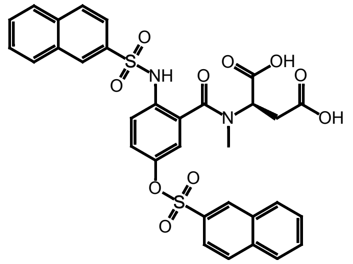

656973
-9.187

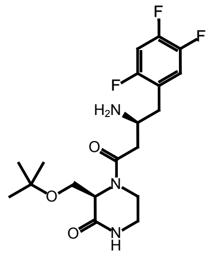

25022354
-9.064

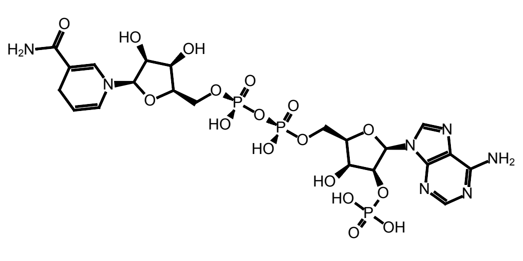

5884
-9.039

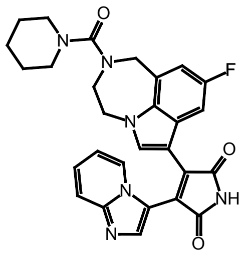

10029385
-9.007

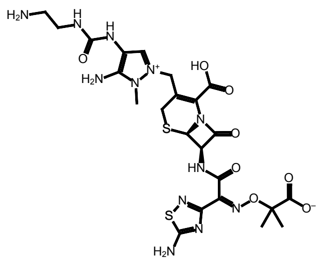

71457955
-9.004

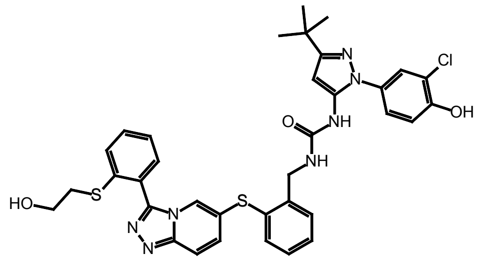

11714580
-8.999

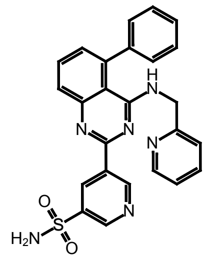

51030730
-8.966

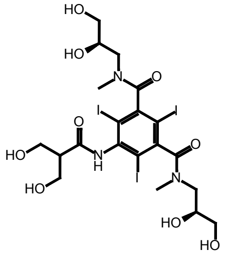

65985
-8.82

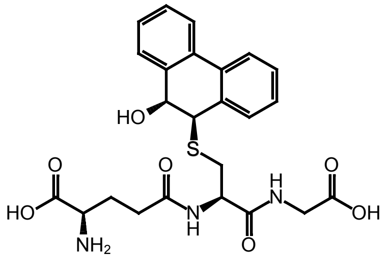

449366
-8.817

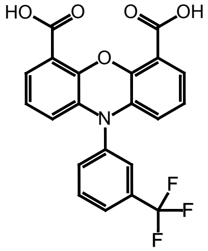

4310
-8.798

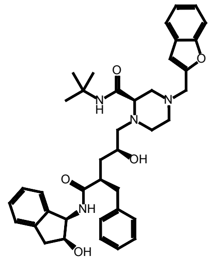

5481481
-8.759

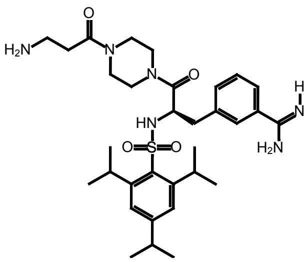

5289531
-8.742

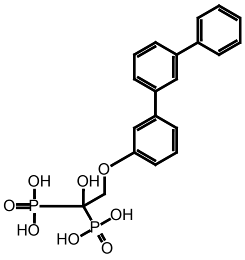

16122556
-8.723

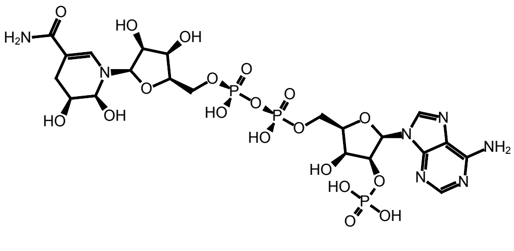

447659
-8.708

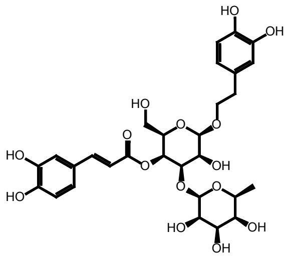

5281800
-8.7

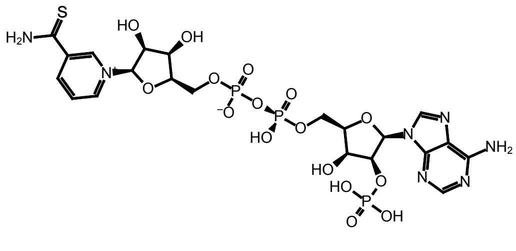

5289437
-8.676

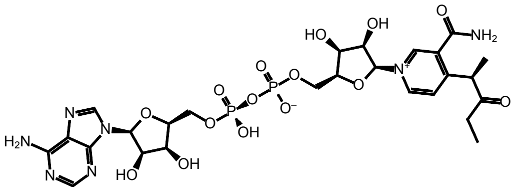

17754101
-8.67

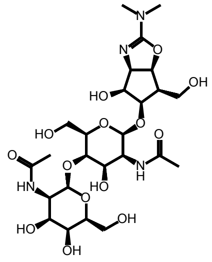

119339
-8.669

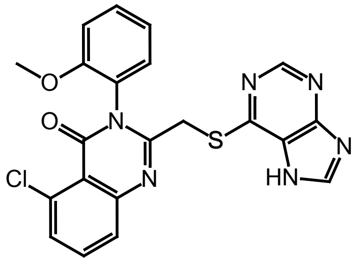

6852165
-8.625

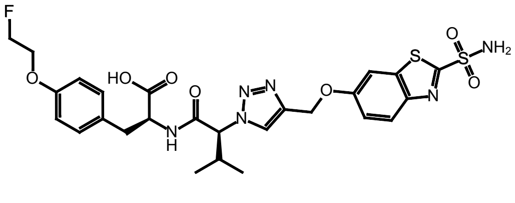

59552698
-8.625

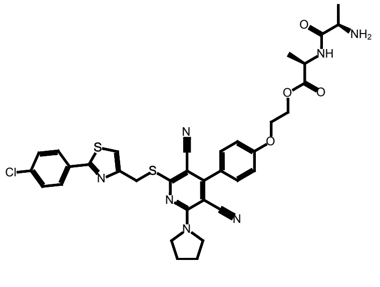

56848985
-8.591

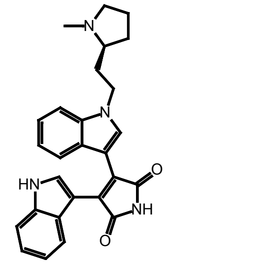

448943
-8.577

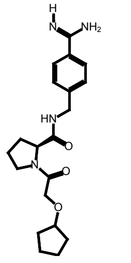

24963035
-8.562

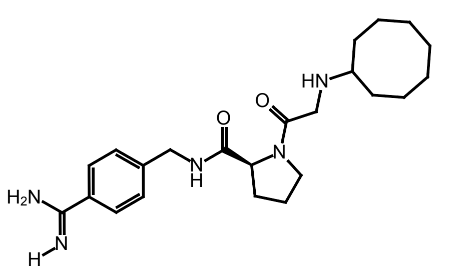

46937030
-8.556

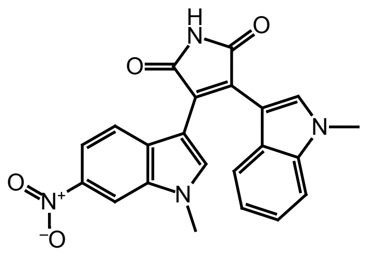

5327686
-8.554

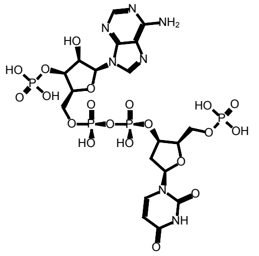

448108
-8.552

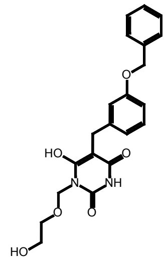

5287741
-8.548

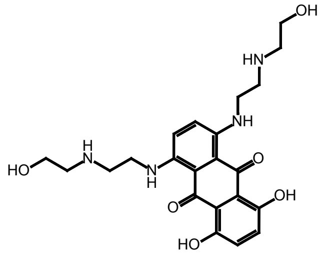

4212
-8.515

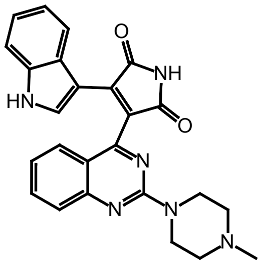

10296883
-8.507

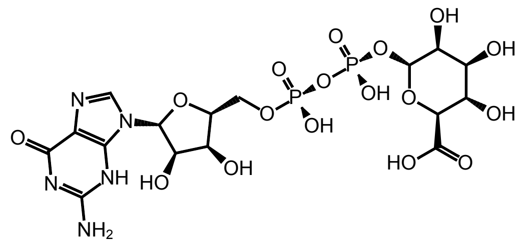

447152
-8.506

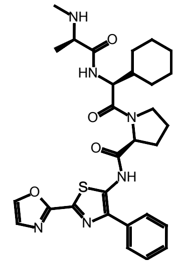

71600094
-8.499

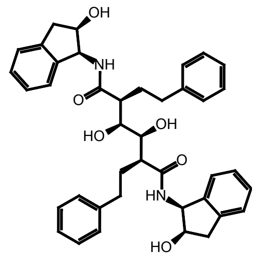

449129
-8.493

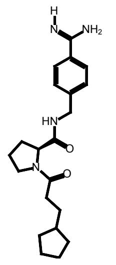

24963037
-8.478

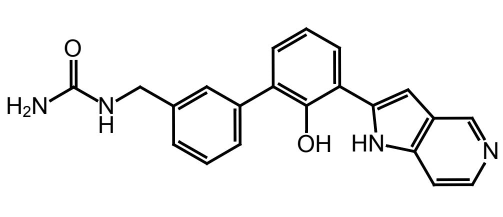

15942655
-8.468

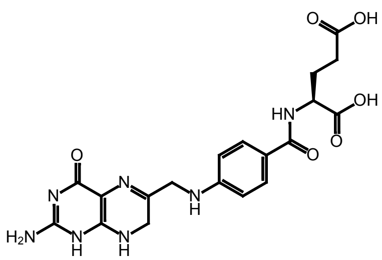

98792
-8.455

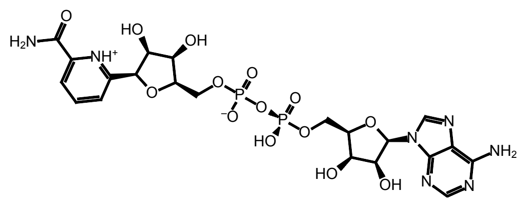

5289104
-8.452

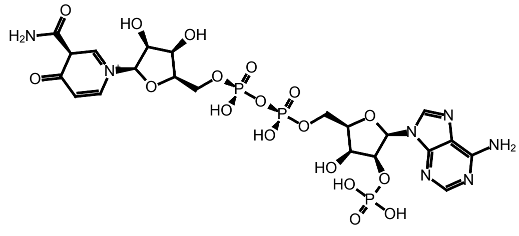

49867432
-8.438

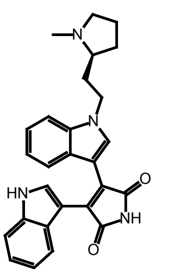

448642
-8.437

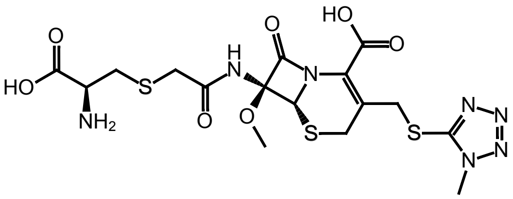

71141
-8.428

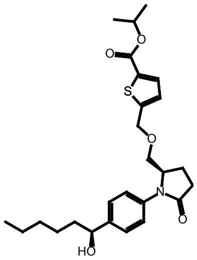

46902081
-8.418

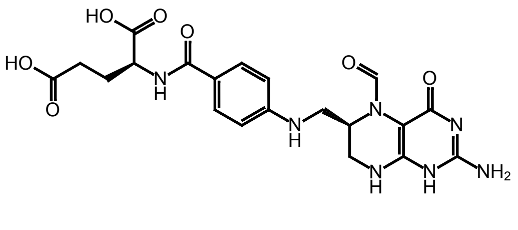

149436
-8.41

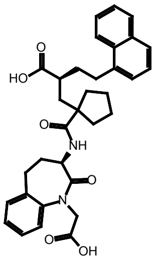

11215055
-8.408

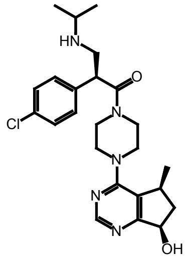

24788740
-8.391

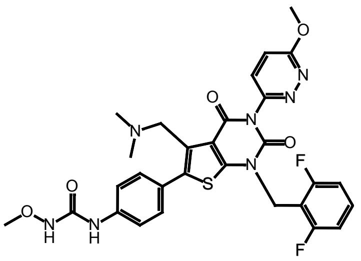

10348973
-8.389

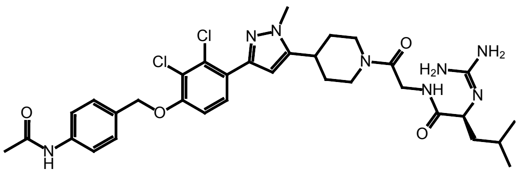

656989
-8.385

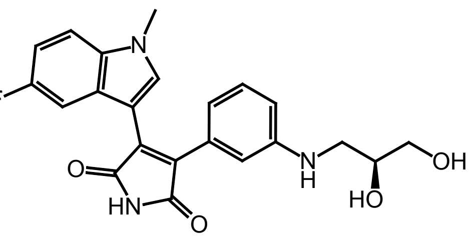

448238
-8.382

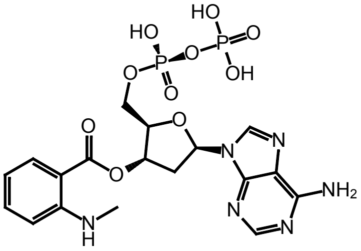

5288821
-8.376

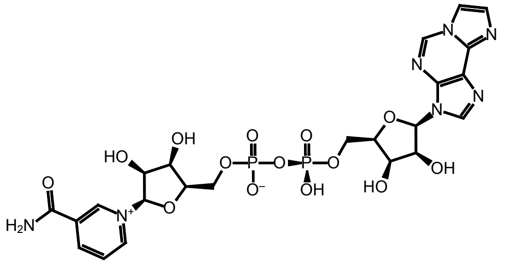

170119
-8.369

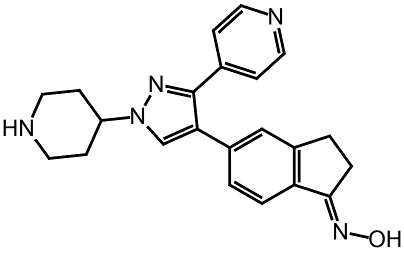

11653652
-8.367

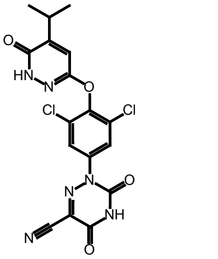

15981237
-8.358

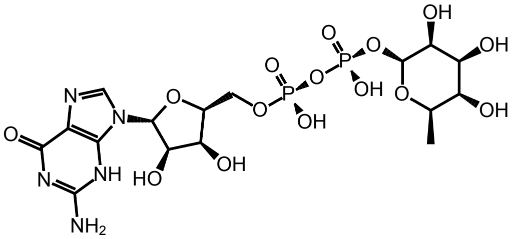

439912
-8.342

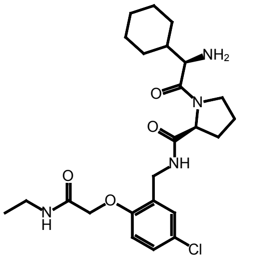

448677
-8.32

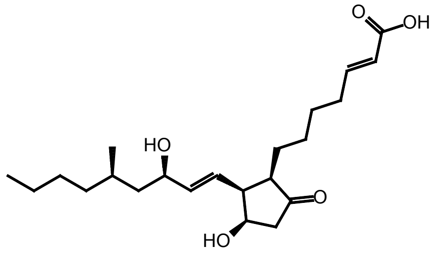

6438378
-8.314

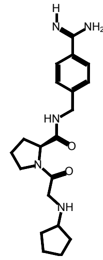

23629654
-8.311

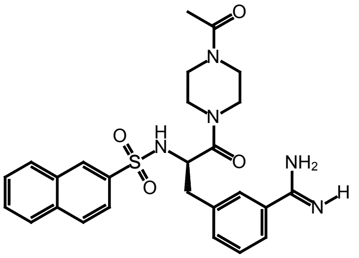

178051
-8.31

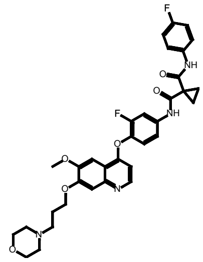

42642645
-8.307

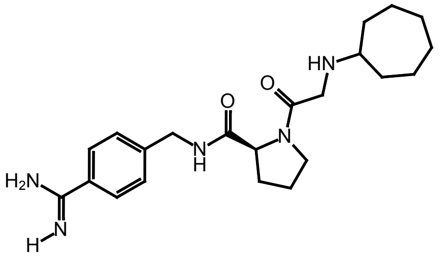

25113614
-8.307

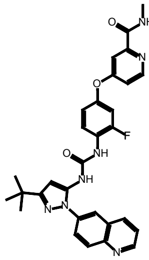

25066467
-8.296

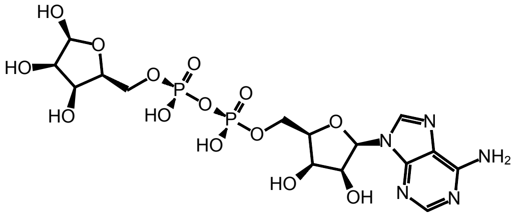

447048
-8.296

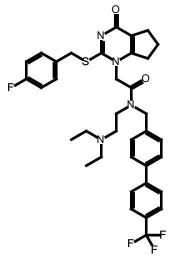

9939609
-8.292

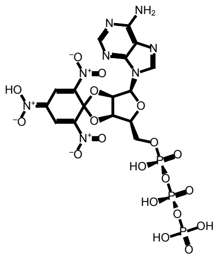

644358
-8.289

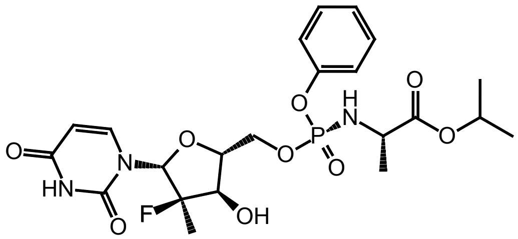

45375808
-8.282

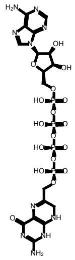

6323200
-8.277

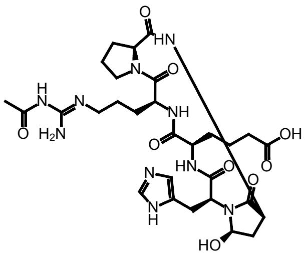

449123
-8.267

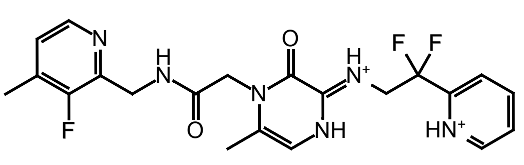

6323250
-8.262

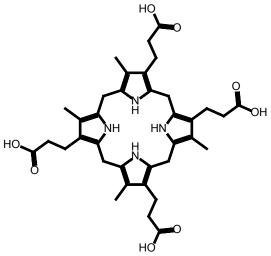

321
-8.256

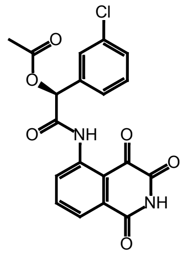

46937155
-8.244

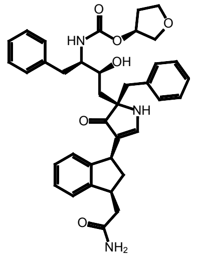

5459370
-8.235

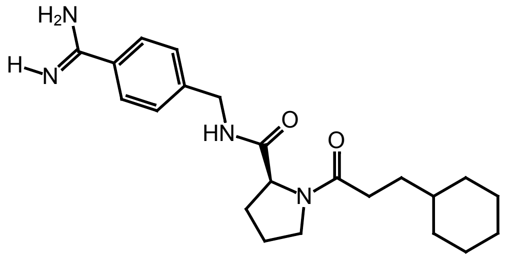

25134248
-8.233

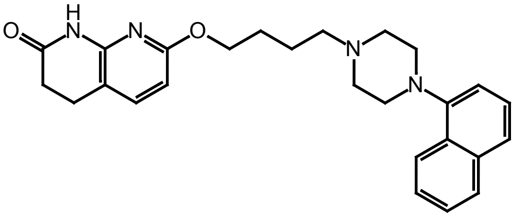

11697676
-8.228

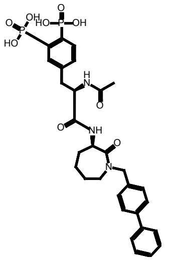

447527
-8.213

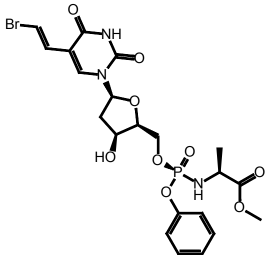

6440764
-8.212

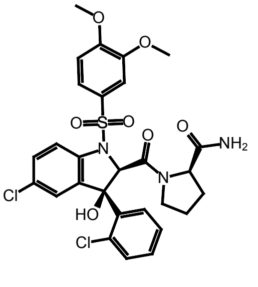

60943
-8.209

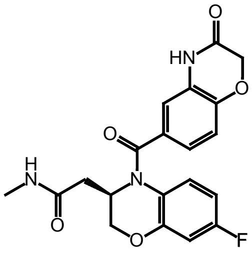

118599727
-8.205

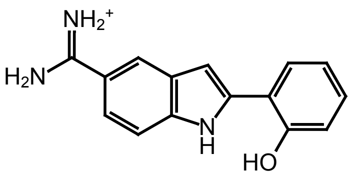

1507
-8.204

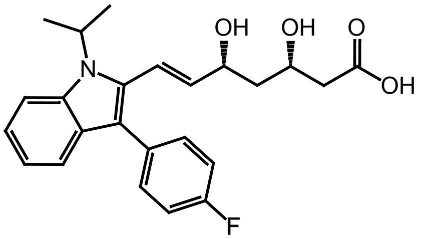

1548972
-8.201

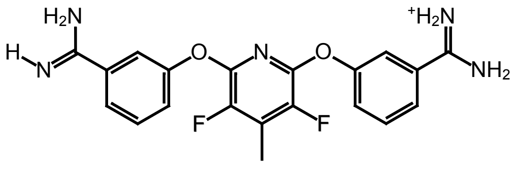

4470378
-8.189

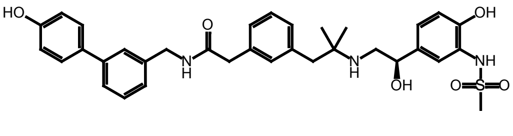

11505444
-8.184

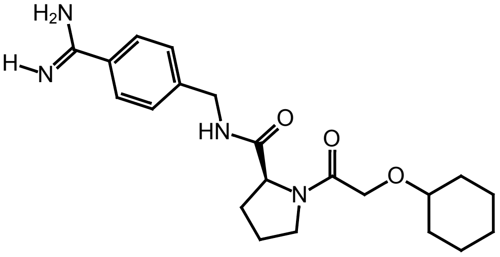

24963036
-8.157

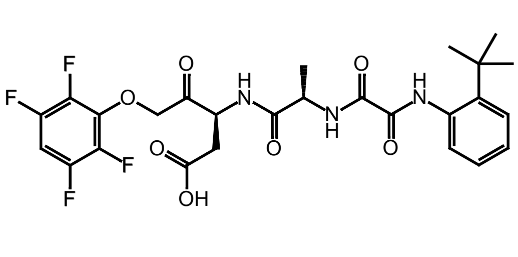

12000240
-8.152

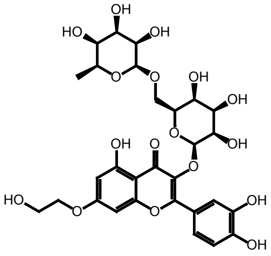

9852585
-8.146

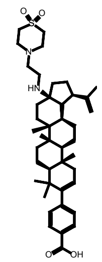

60152109
-8.145

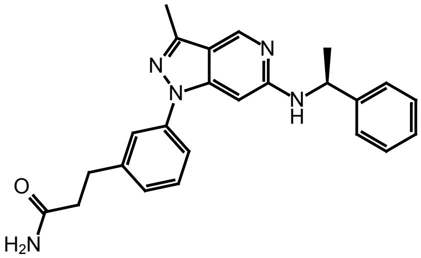

24941249
-8.143

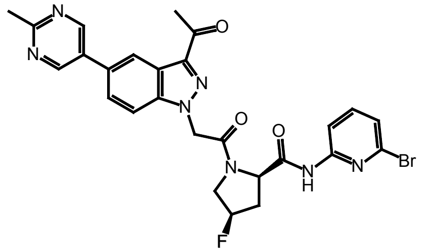

118323590
-8.141

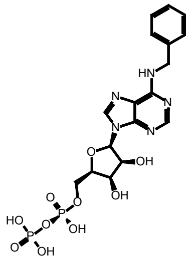

446795
-8.138

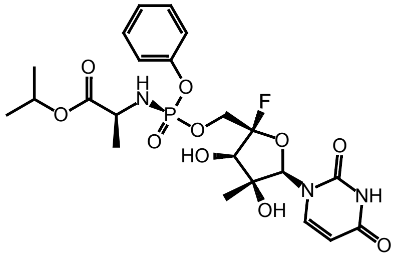

118596336
-8.136

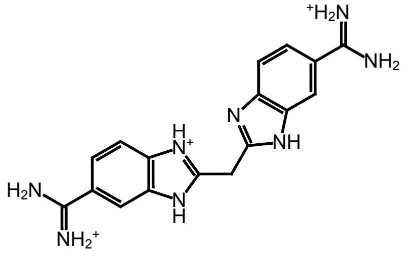

6398417
-8.13

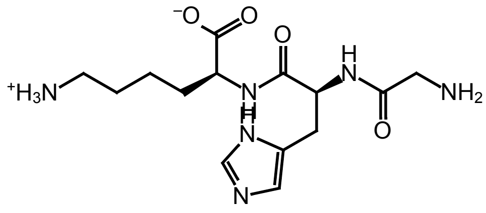

9862773
-8.126

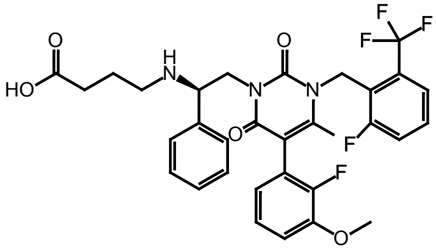

11250647
-8.125

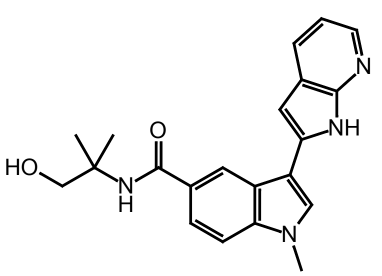

9968957
-8.114

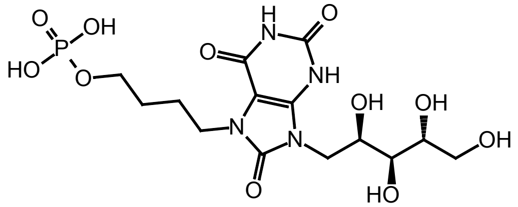

657027
-8.112

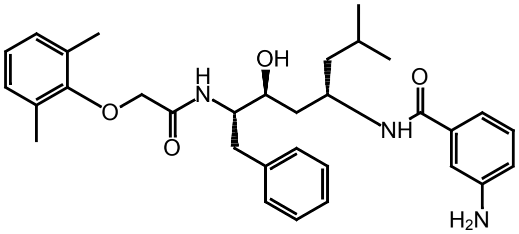

446912
-8.106

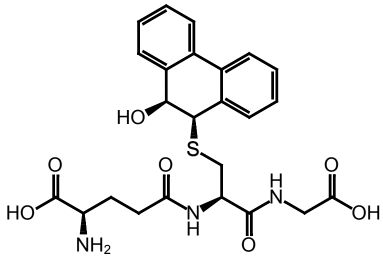

444461
-8.099

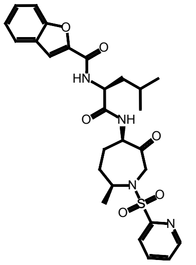

6918602
-8.094

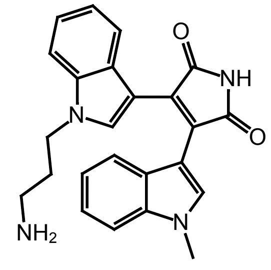

2403
-8.087

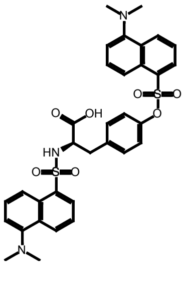

446468
-8.073

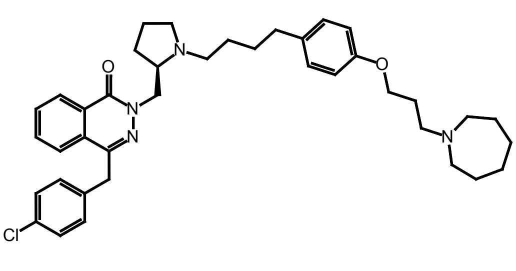

17747460
-8.068

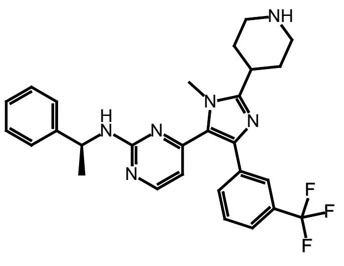

447721
-8.064

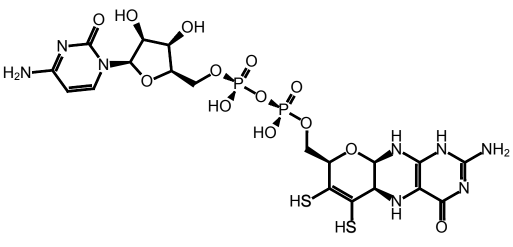

4369128
-8.057

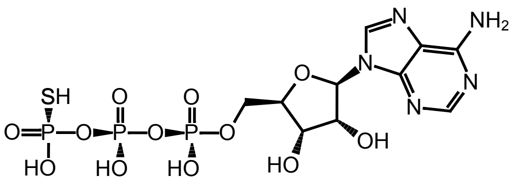

444377
-8.057

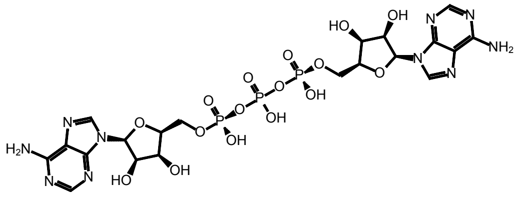

165381
-8.055

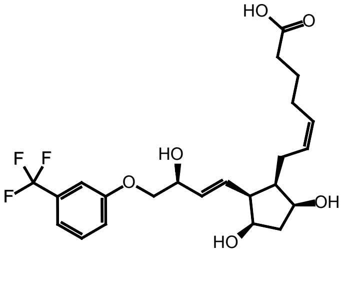

5311100
-8.053

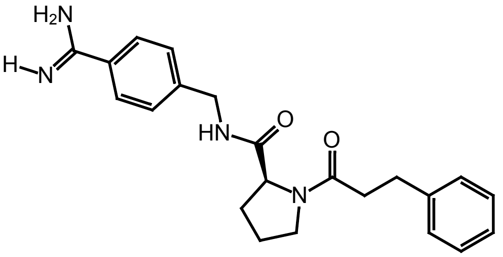

25113615
-8.048

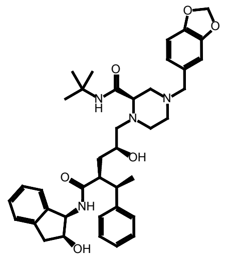

446636
-8.045

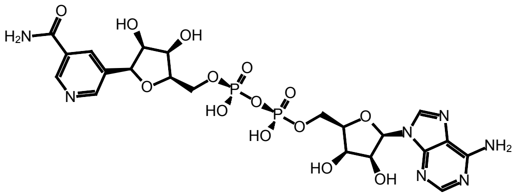

444215
-8.043

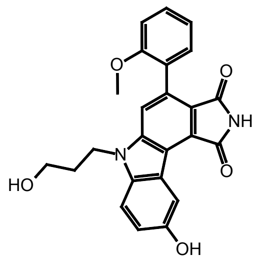

10364585
-8.037

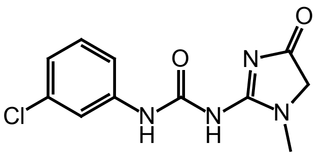

162834
-8.035

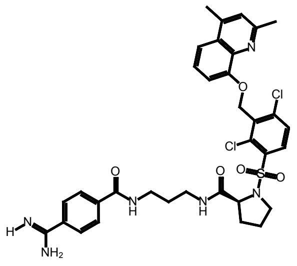

9831652
-8.035

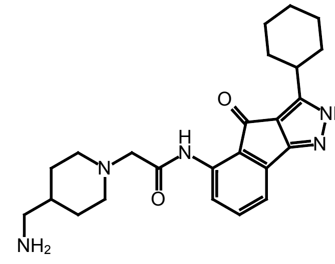

5288017
-8.034

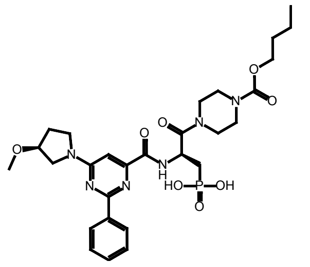

59534142
-8.028

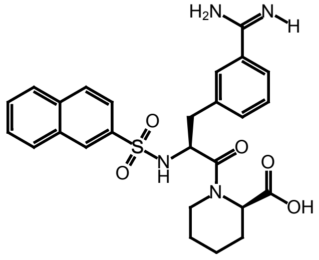

446604
-8.027

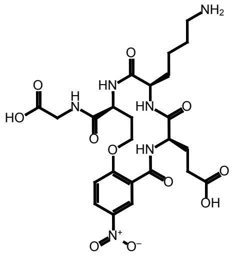

9808372
-8.02

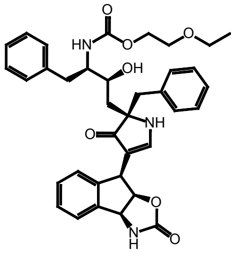

5459358
-8.02

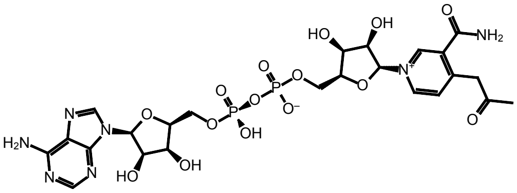

5288882
-8.019

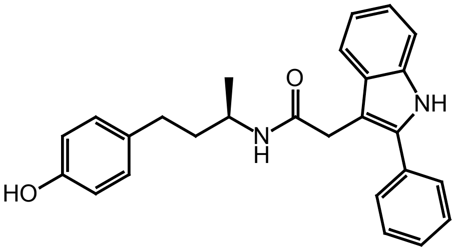

16750040
-8.018

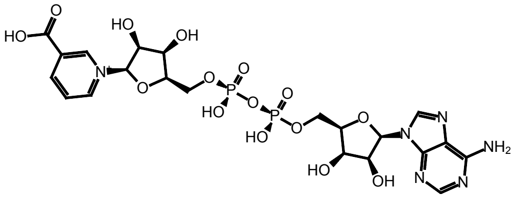

165491
-8.018

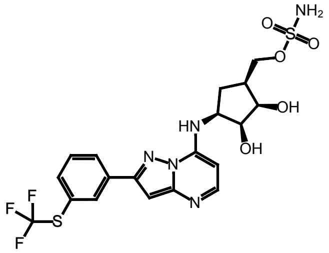

71715374
-8.006

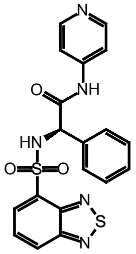

16214776
-8.005
