# Supplementary material for: In Silico Insights into the SARS CoV-2 Main Protease Suggest NADH Endogenous Defences in the Control of the Pandemic Coronavirus Infection
Source: Viruses. 2020 Jul 26;12(8):805. doi: 10.3390/v12080805 (PMC7472248; doi:10.3390/v12080805)
Supplement: Supplementary file 1 [file viruses-12-00805-s001.zip › Supplementary_Material-S2.pdf]

Research Article

In silico insights on SARS Cov-2 Main Protease suggest NADH endogenous defences in the control of the pandemic coronavirus infection

Annamaria Martorana, Carla Gentile, Antonino Lauria*

Dipartimento di Scienze e Tecnologie Biologiche Chimiche e Farmaceutiche - University of Palermo, Viale delle Scienze – Ed. 17 - I-90128 Palermo, Italy.

*Correspondence: antonino.lauria@unipa.it; Phone +39 091238-96818

Supplementary material S2

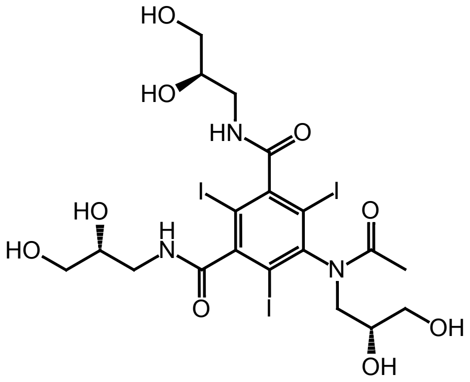

3730
Iohexol

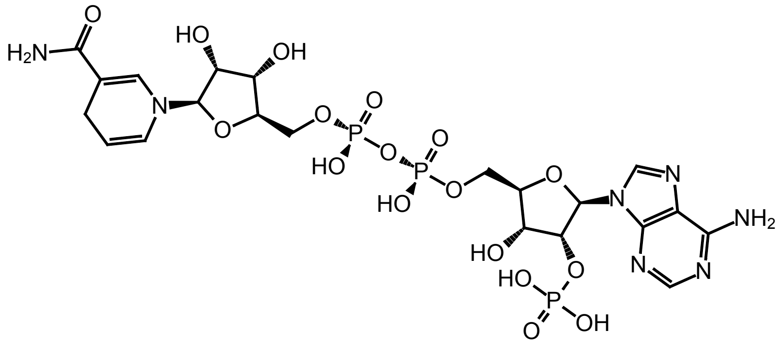

5884
Nadph Dihydro-Nicotinamide-Adenine-Dinucleotidephosphate

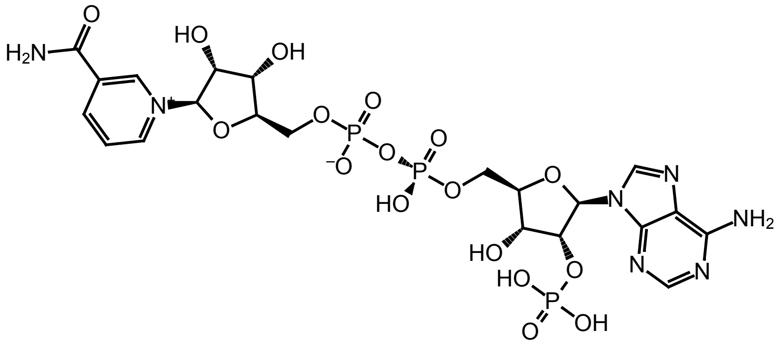

5885
Nicotinamide adenine dinucleotide phosphate

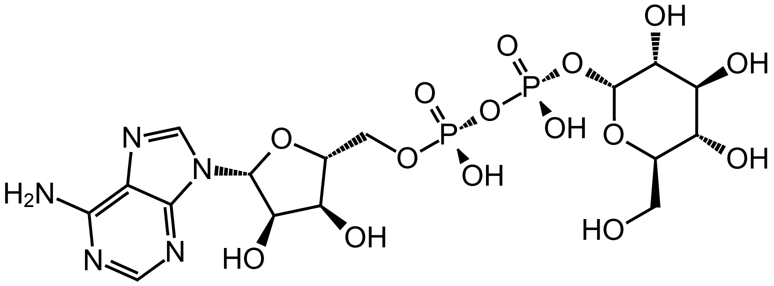

16500
Adenosine-5'-Monophosphate Glucopyranosyl-Monophosphate Ester

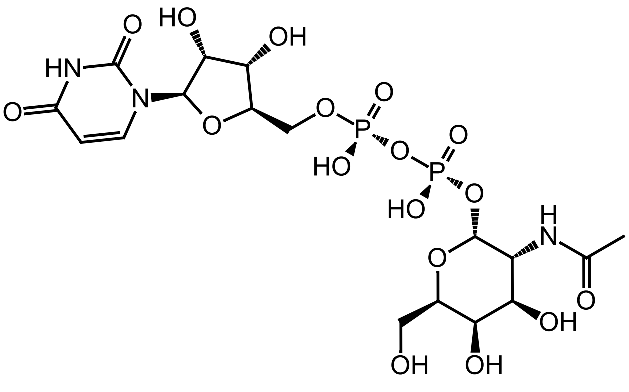

23700
Uridine-Diphosphate-N-Acetylgalactosamine

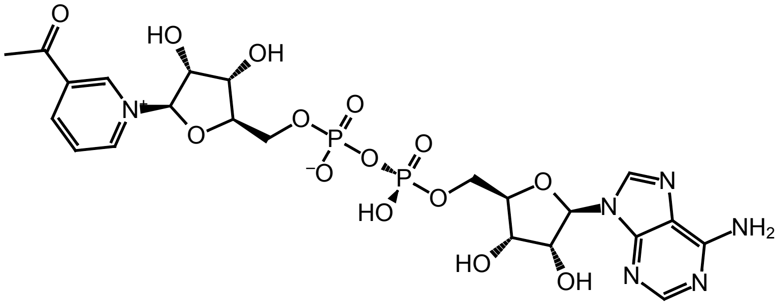

123926
3-Acetylpyridine Adenine Dinucleotide

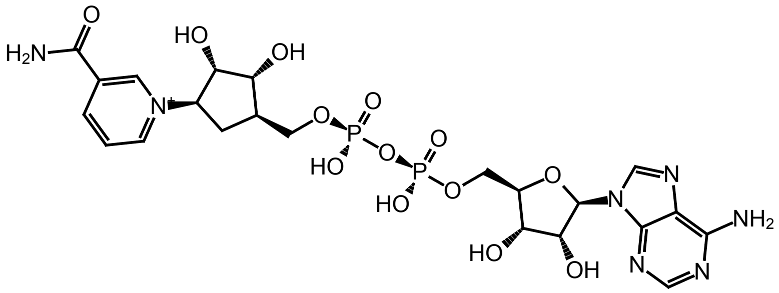

163884
Carba-nicotinamide-adenine-dinucleotide

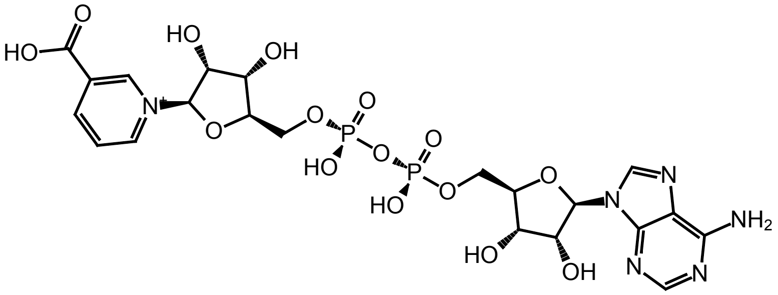

165491
Deamido-Nad+

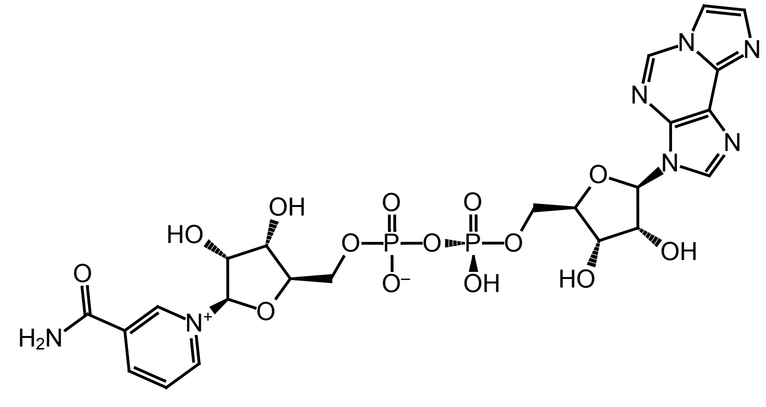

170119
Etheno-NAD

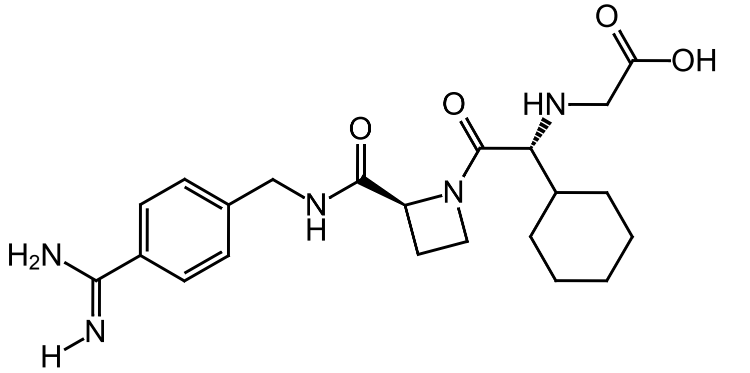

183797
Melagatran

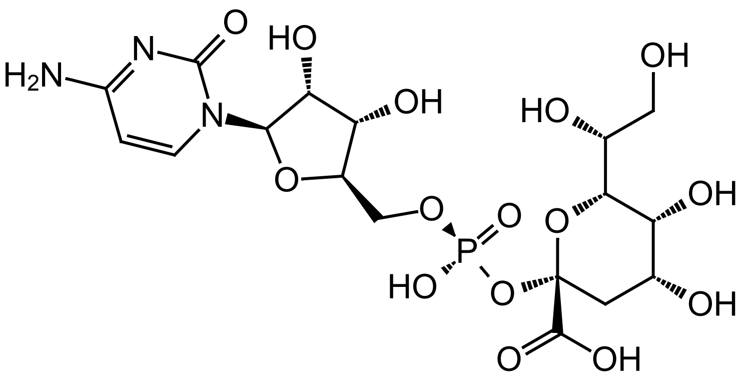

445888
Cmp-2-Keto-3-Deoxy-Octulosonic Acid

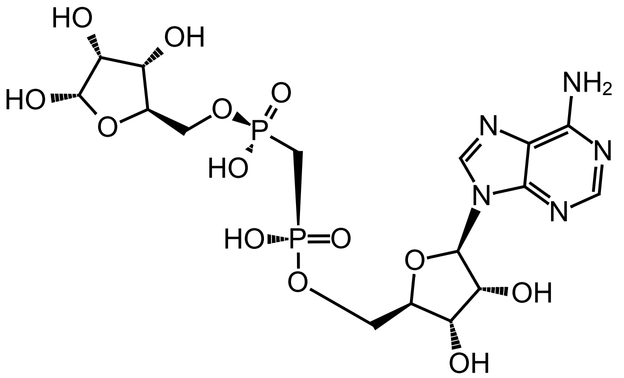

446724
AMPCPR

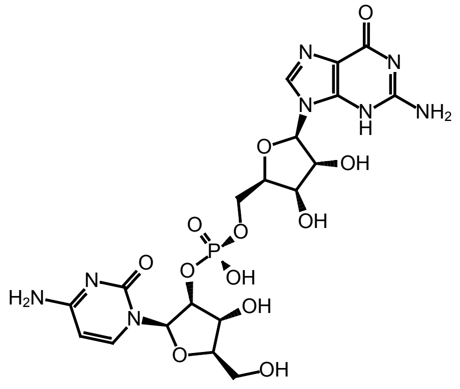

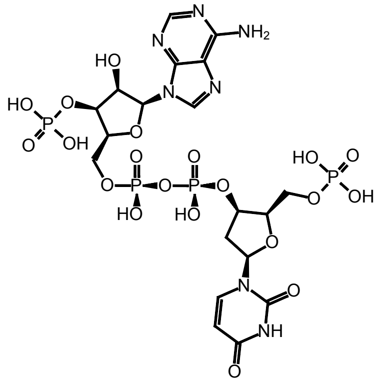

447657
Cytidyl-2'-5'-phospho-guanosine

448108
Adenylate-3'-phosphate-[[2'-deoxy-uridine-5'-phosphate]-3'-phosphate]

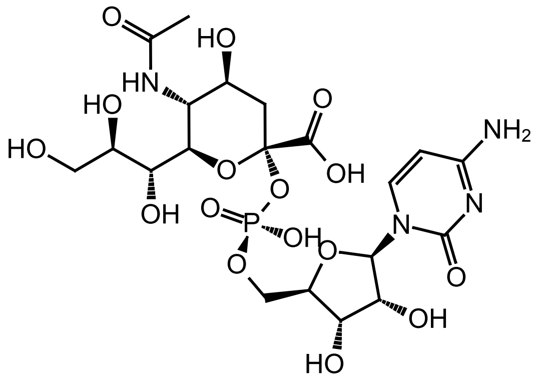

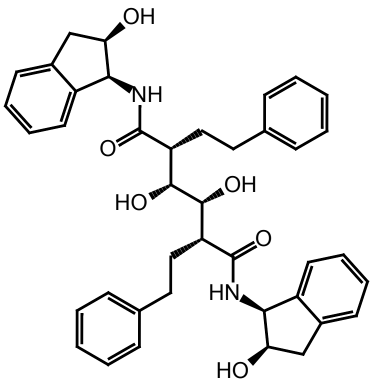

448209
Cytidine-5'-Monophosphate-5-N-Acetylneuraminic Acid

449129
(2R,3R,4R,5R)-3,4-Dihydroxy-N,N'-bis[(1S,2R)-2-hydroxy-2,3-dihydro-1H-inden-1-yl]-2,5-bis(2-phenylethyl)hexanediamide

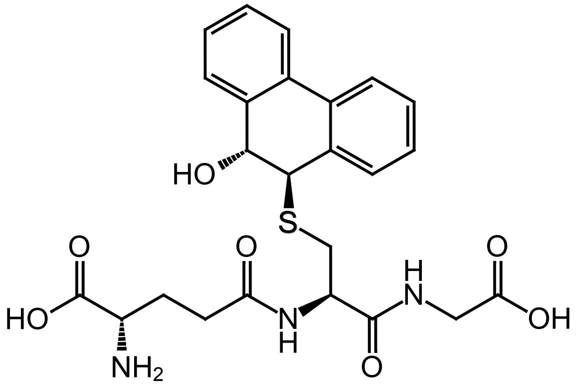

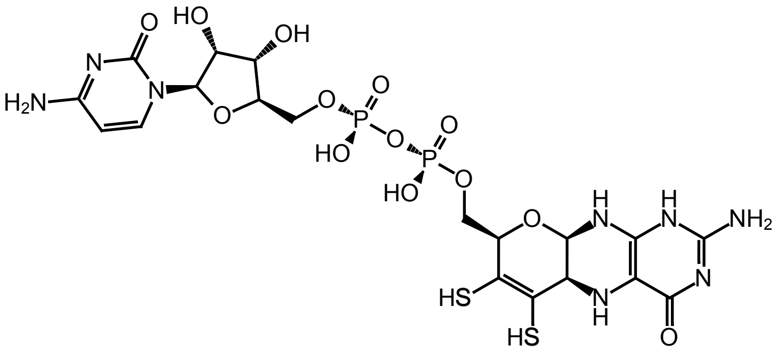

449366
(9R,10R)-9-(S-glutathionyl)-10-hydroxy-9,10-dihydrophenanthrene

4369128
Pterin Cytosine Dinucleotide

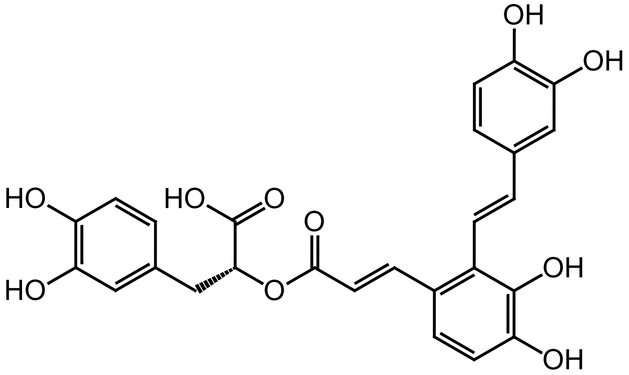

5281793
Salvianolic acid A

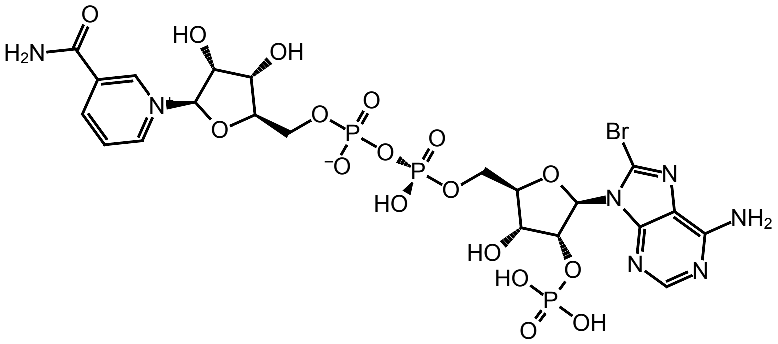

5288989
Nicotinamide 8-Bromo-Adenine Dinucleotide Phosphate

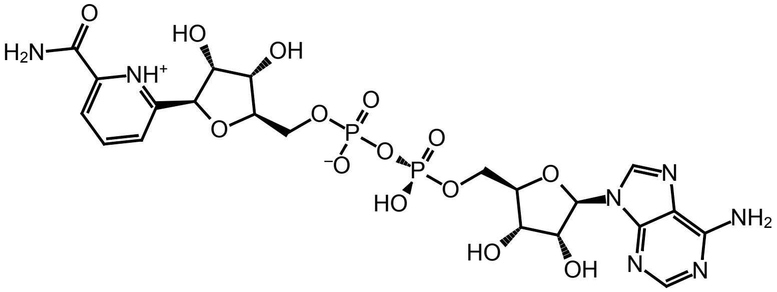

5289104
Cpad

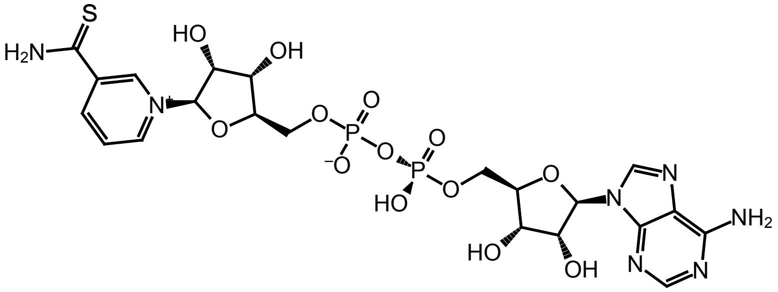

5289382
Thionicotinamide-Adenine-Dinucleotide

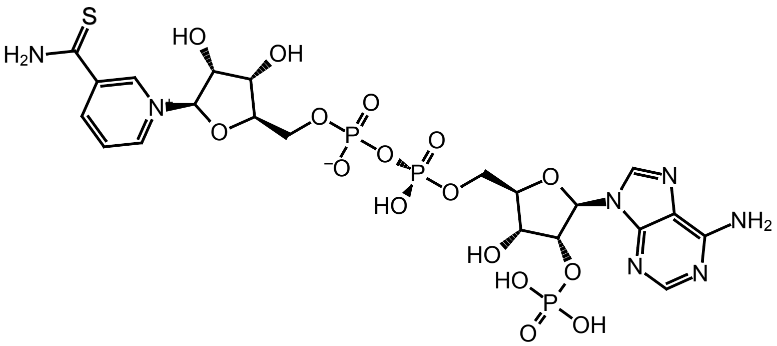

5289437
7-thionicotinamide-adenine-dinucleotide phosphate

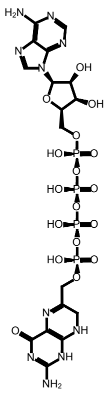

6323200
6-(Adenosine Tetraphosphate-Methyl)-7,8-Dihydropterin

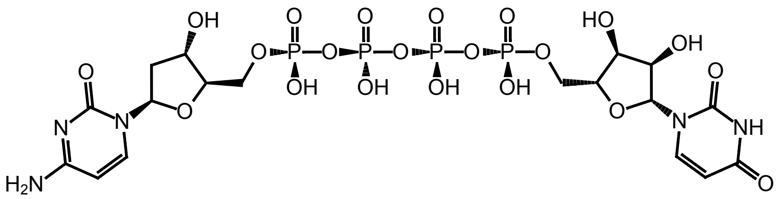

9875516
Denufosol

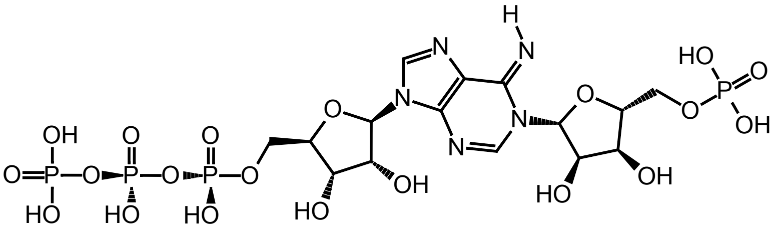

16019963
1-(5-phospho-D-ribosyl)-ATP

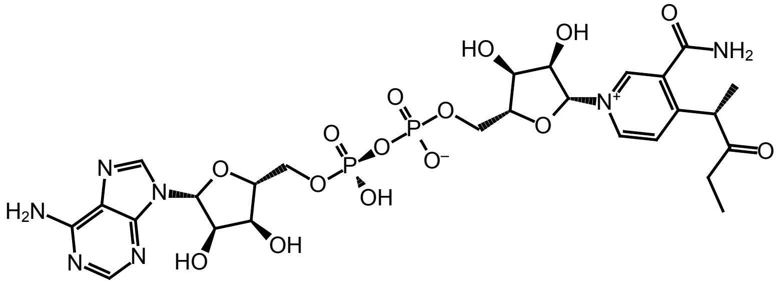

17754101
Nicotinamide adenine dinucleotide 3-pentanone adduct

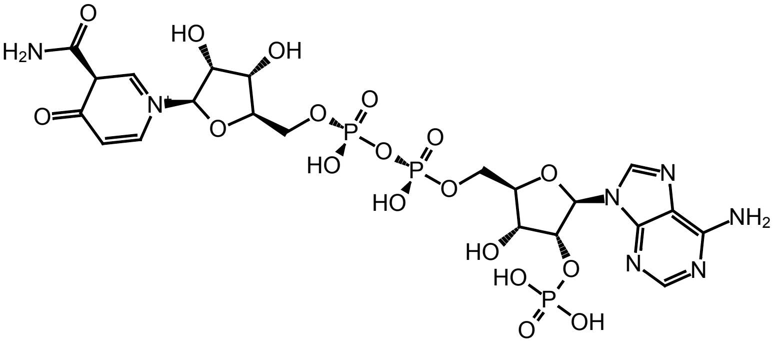

49867432
4-Oxo-nicotinamide-adenine dinucleotide phosphate
